# Central Giant Cell Granuloma of the Posterior Maxilla

**DOI:** 10.7759/cureus.92340

**Published:** 2025-09-15

**Authors:** Irukulla Venkata Krishna, G. Venkateswara Reddy, Mohammed Darain Shahid, G.Siva Prasada Reddy, Godvine Sarepally

**Affiliations:** 1 Oral and Maxillofacial Surgery, Panineeya Mahavidyalaya Institute of Dental Sciences and Research Centre, Hyderabad, IND

**Keywords:** central giant cell granuloma, oral and maxillofacial surgery, oral lesion, oral pathology, oral surgery

## Abstract

Central giant cell granuloma (CGCG) is an uncommon, benign, proliferative intraosseous lesion of the jaws with an uncertain etiology. It is more frequently reported in the mandible, whereas maxillary involvement is relatively rare. We present the case of a 30-year-old female patient with a swelling in the right posterior maxilla that had gradually increased in size over a year. Clinical examination revealed a firm, tender, and expansile lesion, while radiographic evaluation demonstrated a well-defined radiolucent lesion associated with the maxillary posterior teeth. Histopathological analysis confirmed the diagnosis of a CGCG. The lesion was surgically treated with enucleation, curettage, and peripheral ostectomy under general anesthesia. This case underscores the significance of correlating clinical, radiological, and histopathological features for the accurate diagnosis of maxillary lesions and highlights surgical management as an effective treatment modality for CGCG.

## Introduction

A central giant cell granuloma (CGCG) is a benign, non-neoplastic intraosseous lesion of the jaws, first described by Jaffe in 1953 as a “giant cell reparative granuloma” to distinguish it from true giant cell tumors of the long bones [1]. Histologically, it is composed of multinucleated giant cells in a fibroblastic stroma with areas of hemorrhage and new bone formation [2]. Although its exact etiology remains unclear, trauma, intraosseous hemorrhage, and reactive processes have been proposed [3].

CGCG represents approximately 7% of all benign jaw lesions and occurs more frequently in children and young adults, with a female predilection [4,5]. The mandible is more commonly affected than the maxilla, and the anterior regions are more commonly affected than the posterior regions [6]. Clinically, lesions range from asymptomatic swellings to aggressive masses that produce pain, cortical expansion, root resorption, or displacement of teeth [7]. Radiographically, CGCG may appear as unilocular or multilocular radiolucencies, sometimes with wispy septations and undulating borders, closely resembling odontogenic cysts or tumors [8].

The biological behavior of CGCG is variable, with some lesions showing slow indolent growth, while others are aggressive and locally destructive [4,9]. Treatment strategies include surgical curettage, resection, and conservative alternatives such as intralesional corticosteroids, calcitonin, interferon-α, and denosumab, although surgical excision with curettage remains the most accepted modality [10].

Here, we present a case of CGCG involving the posterior maxilla in a 30-year-old female patient, emphasizing its clinical presentation, diagnostic evaluation, and surgical management.

## Case presentation

A 30-year-old female patient reported to the Department of Oral and Maxillofacial Surgery, Panineeya Mahavidyalaya Institute of Dental Sciences and Research Centre, Hyderabad, India, with a complaint of swelling in the right upper back tooth region for the past year. The patient reported a gradual increase in the size of the swelling, which was initially small and asymptomatic but later resulted in mild facial asymmetry and discomfort during chewing. Her medical history was significant for supraventricular tachycardia during pregnancy, which had been successfully managed. She was not on any long-term medications, had no known drug allergies, and was lactating at the time of presentation.

**Figure 1:**
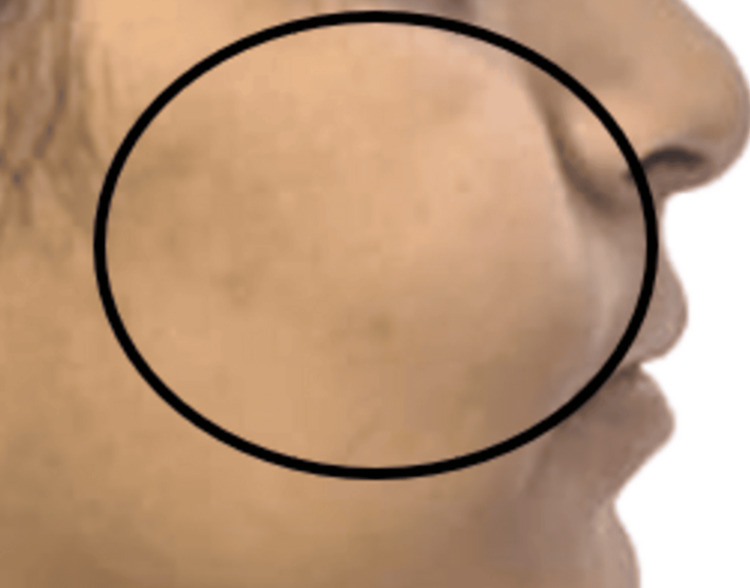
Extraoral image of the patient showing the swelling in the right middle third of the face

On extraoral examination (Figure 1), a diffuse swelling measuring approximately 3 × 3 cm was evident on the right middle third of the face which extended anteroposteriorly from the ala of the nose to a vertical line through the outer canthus of the eye, and superoinferiorly from a point 3 cm below the infraorbital rim to a line connecting the corner of the mouth with the tragus of the ear.

The swelling was firm to hard in consistency, mildly tender, and not fixed to the underlying bone.

**Figure 2:**
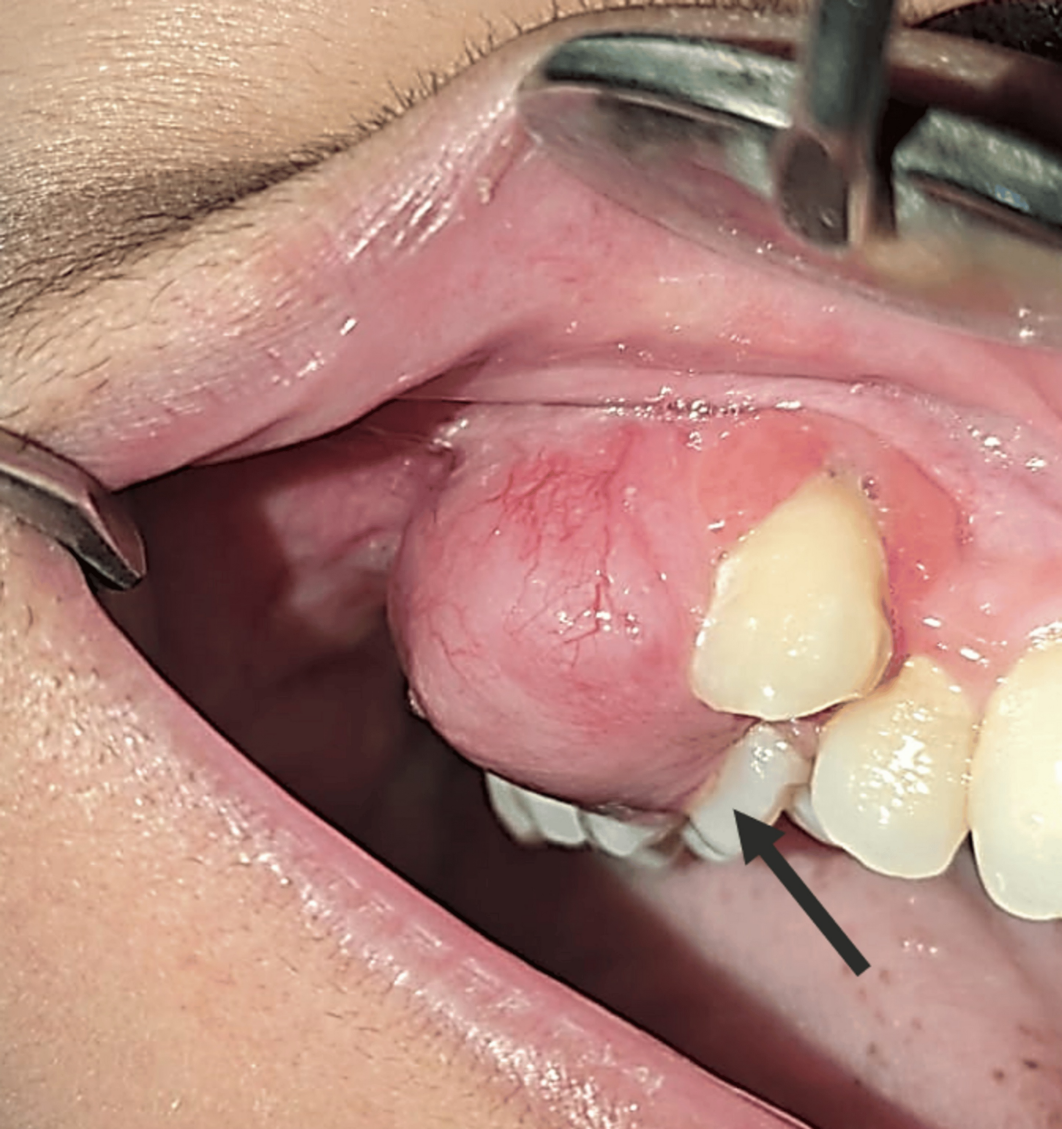
Intraoral image of the patient showing a solitary sessile gingival swelling

Intraoral examination (Figure 2) revealed a solitary sessile gingival swelling involving the marginal, attached, and interdental gingiva in the region of teeth 13 to 16.

The lesion measured approximately 3 × 2 cm, appeared pale pink in color, and showed surface vascular dilatations. Buccal and palatal cortical expansion was evident, resulting in vestibular obliteration. Grade I mobility was present in teeth 13 and 14, and teeth 15 and 16 were found to be nonvital.

**Figure 3:**
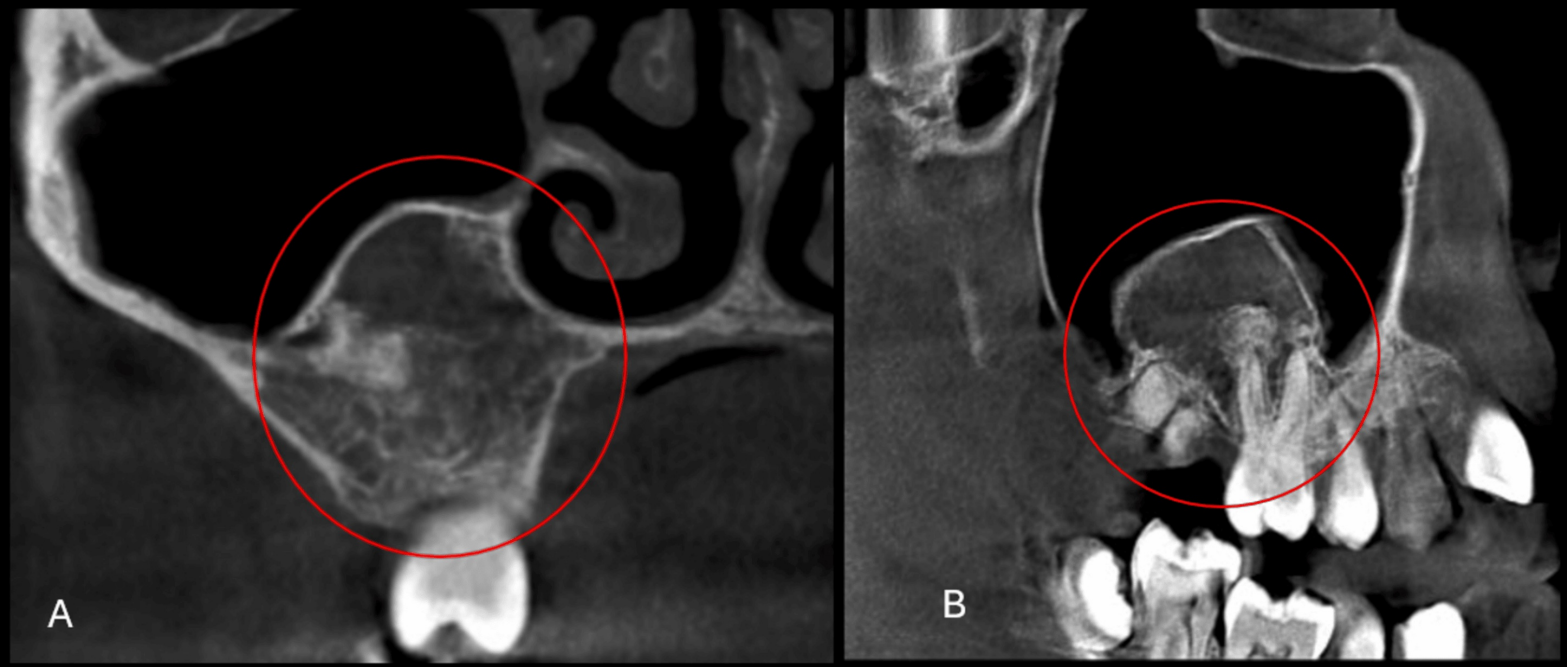
Cone-beam computed tomography (CBCT) images A) Coronal section of the CBCT showing the lesion extending superoinferiorly from the floor of the maxillary sinus to the alveolar crest. B) Sagittal section of the CBCT showing the lesion extending anteroposteriorly from the distal aspect of tooth 13 to the region of tooth 17.

Cone-beam computed tomography (CBCT) revealed a well-defined radiolucent lesion with patchy areas of increased density, suggestive of calcifications. On coronal section of the CBCT, the lesion was seen extending superoinferiorly from the floor of the maxillary sinus to the alveolar crest, and in the sagittal section of the CBCT, the lesion was seen extending anteroposteriorly from the distal aspect of tooth 13 to the region of tooth 17 (Figure 3).

The lesion exhibited characteristic wispy septations, undulating borders, cortical expansion with thinning, and displacement of the sinus floor. Root resorption of teeth 13-16 was also observed.

**Figure 4:**
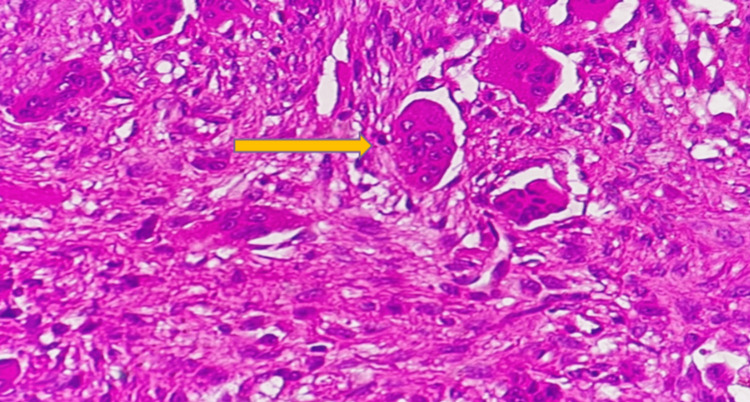
Photomicrograph showing multinucleated giant cells arranged in clusters Hematoxylin and Eosin (H&E) stain, original magnification ×40.

An incisional biopsy was performed, and histopathological examination of hematoxylin and eosin-stained sections revealed multinucleated giant cells arranged in clusters, unevenly distributed within a spindle-shaped fibroblastic stroma (Figure 4).

Foci of hemorrhage, hemosiderin deposition, and trabeculae of reactive new bone formation were also noted. These features confirmed the diagnosis of CGCG.

**Figure 5:**
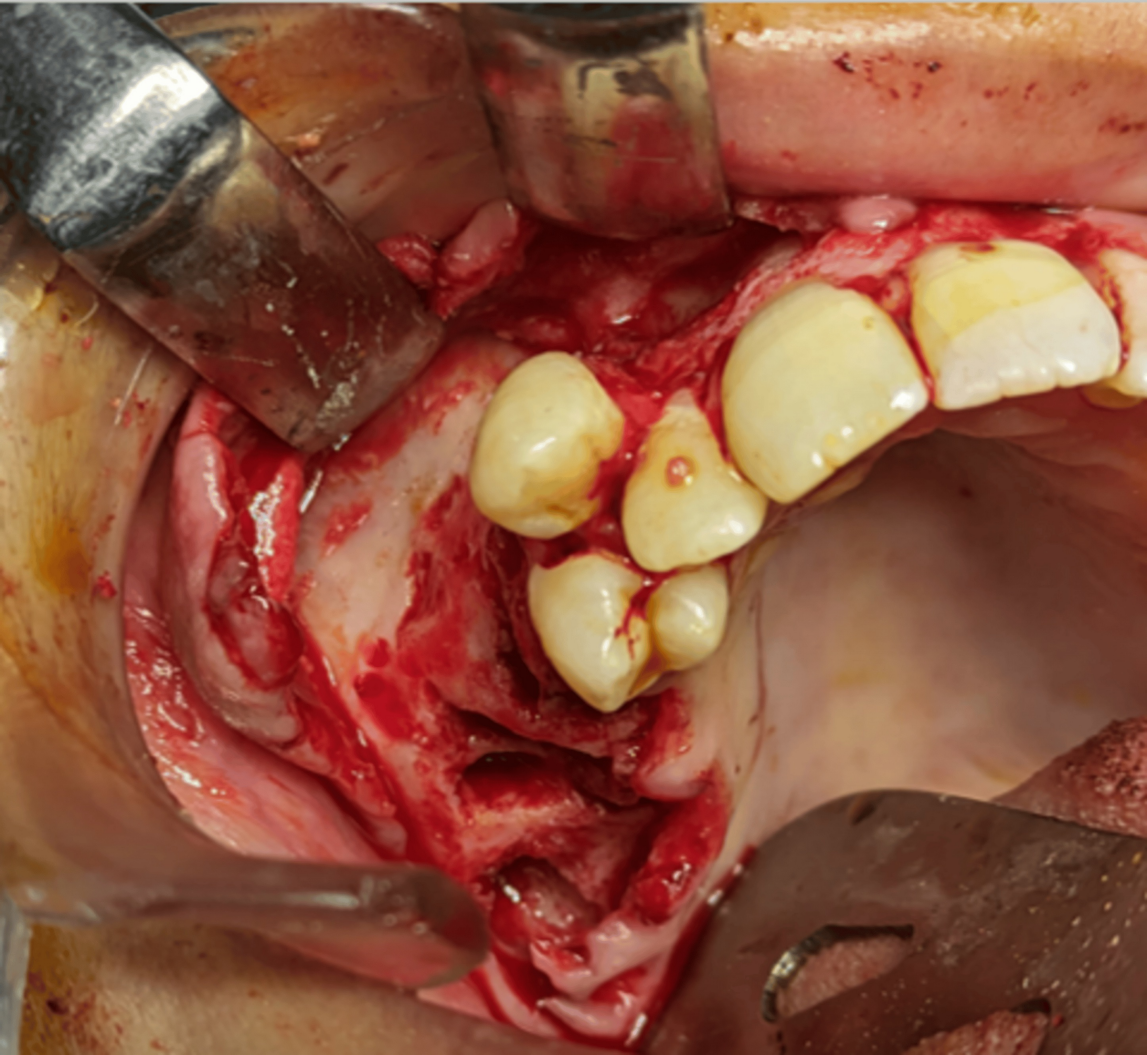
Intraoperative image after enucleation and curettage followed by peripheral ostectomy

Definitive surgical management was performed under general anesthesia. A crevicular incision was made from teeth 11 to 17, and a full thickness mucoperiosteal flap was elevated. Teeth 15, 16, and 17, which were directly involved in the lesion, were extracted. The lesion was completely enucleated and curetted through the extraction sockets, followed by peripheral ostectomy of the surrounding bone margins using a round bur to minimize recurrence (Figure 5).

Hemostasis was achieved, and the surgical site was thoroughly irrigated with saline before primary closure using 3-0 Truglyde^®️^ absorbable braided sutures (Polyglycolic Acid, Healthium Medtech Limited, Bangalore, India). Postoperative recovery was uneventful.

**Figure 6:**
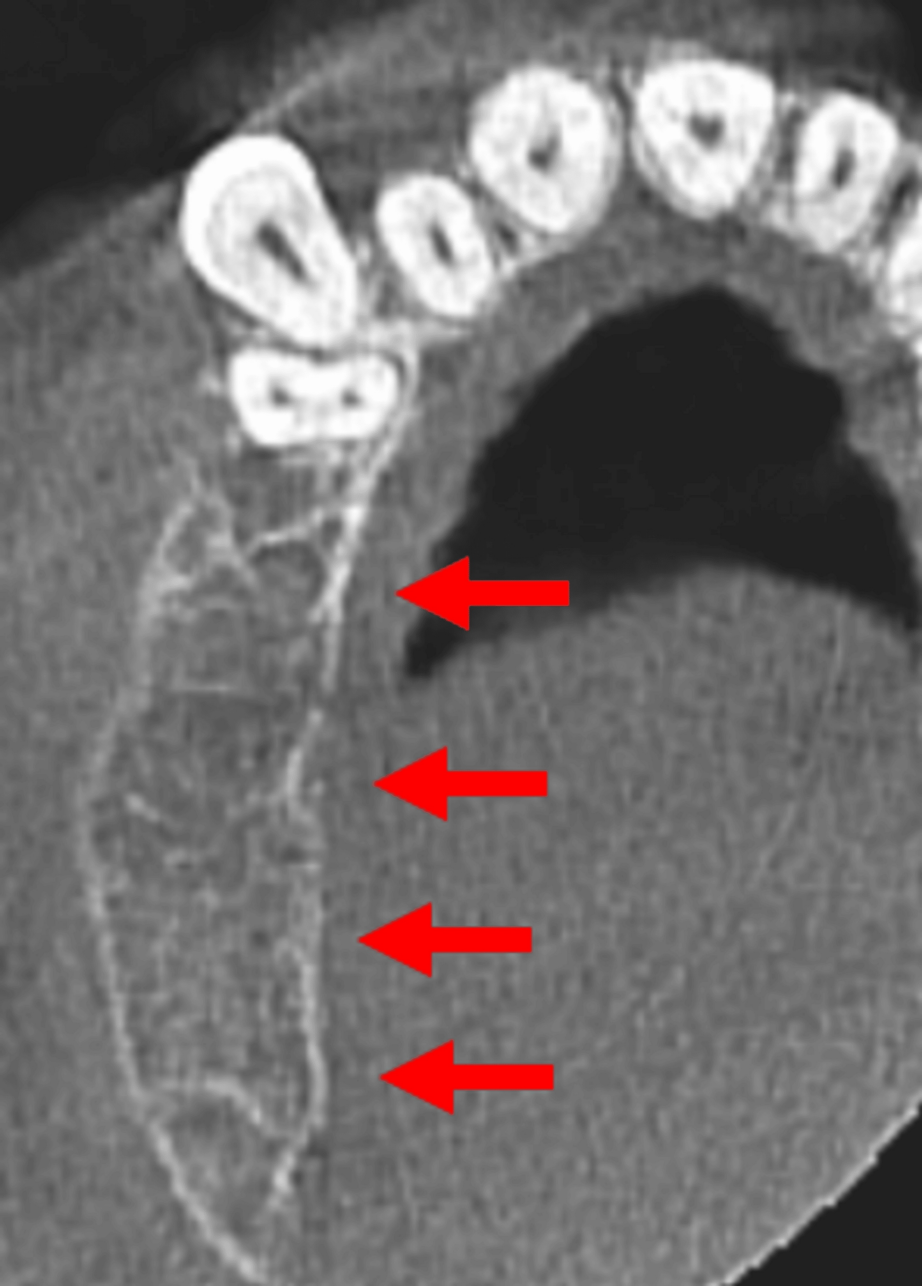
One-year postoperative CBCT section showing satisfactory bone healing with no signs of recurrence in the previously affected right posterior maxilla CBCT: Cone-beam computed tomography.

The patient was followed up regularly, and at the one-year review, intraoral examination revealed healthy mucosal covering with normal gingival margins, good healing of the surgical site, and no evidence of swelling or mucosal irregularities. A follow-up CBCT at one year revealed complete resolution of the lesion with restoration of the normal trabecular bone pattern, intact cortical plates, and no signs of recurrence (Figure 6).

## Discussion

CGCG demonstrates a highly variable biological spectrum, and its clinical significance lies in both its unpredictable behavior and its ability to mimic other odontogenic and non-odontogenic pathologies. In the present case, the lesion involved the posterior maxilla of a 30-year-old female patient, which is less common than the more frequently affected anterior mandible in younger patients [4,6]. Reports indicate that posterior maxillary CGCGs are relatively rare but may have greater clinical relevance because of the thin cortical plates, proximity to the maxillary sinus and orbit, and the potential for extensive destruction before clinical detection [8,9].

Our patient presented with a slowly enlarging, painless swelling with cortical expansion and mobility of the adjacent teeth. These features were consistent with non-aggressive CGCG, as described in prior literature [5,7,11]. Eisenbud et al. classified lesions into aggressive and non-aggressive subtypes, noting that the latter typically present without pain, rapid growth, or cortical perforation, and were associated with a lower recurrence rate following surgery [7]. The absence of pain and soft tissue invasion in our case supported its classification as a non-aggressive lesion.

Radiographically, the radiolucent lesion with wispy septations seen in this case correlated with the classical descriptions by Stavropoulos and Katz and Kaffe et al. [6,8]. However, the radiological features alone were not pathognomonic, as similar appearances can be found in ameloblastoma, odontogenic myxoma, and ossifying fibroma. This emphasizes the necessity of histopathological confirmation, particularly for lesions of the posterior maxilla, where differential diagnoses are broad and management protocols vary widely [9,12].

Histopathological analysis in our case revealed multinucleated giant cells irregularly distributed in a spindle-shaped fibroblastic stroma, consistent with the classical description of CGCG [2,4,13]. Immunohistochemical studies in prior research have demonstrated that these giant cells share phenotypic similarities with osteoclasts, supporting the hypothesis that CGCG represents reactive osteoclastic proliferation [2]. These findings reinforce the view that CGCG is not a true neoplasm but rather a reactive lesion with variable growth potential.

Management of CGCG remains one of the most debated aspects of its clinical care because of its unpredictable biologic behavior and variable recurrence rates. Conservative surgical excision with curettage and peripheral ostectomy is still regarded as the gold standard for localized, non-aggressive lesions, with reported recurrence rates ranging between 15-20% [7,10,12]. More radical resection is reserved for aggressive or recurrent cases but carries greater morbidity, particularly in the maxilla where functional and esthetic considerations are critical [10,11,14]. In our case, complete excision with curettage was sufficient, and no recurrence was observed at the one-year follow-up, consistent with the outcomes reported for non-aggressive variants [13,15].

Alternative nonsurgical modalities, such as intralesional corticosteroids, calcitonin, interferon-α, and more recently denosumab, have been explored to avoid the morbidity of surgery in aggressive, recurrent, or pediatric cases [16, 17]. While these therapies have shown promise, they require prolonged treatment and close monitoring, and their long-term efficacy remains uncertain [17]. Thus, surgery continues to remain the most predictable treatment for accessible lesions, as demonstrated in the present case.

The key learning point from this case lies in the importance of correlating clinical, radiographic, and histopathological findings to establish a definitive diagnosis, particularly in unusual sites such as the posterior maxilla. Furthermore, structured follow-up with radiographic monitoring is essential, as most recurrences occur within the first two years [7,14]. Our patient demonstrated complete bone healing at one year, with CBCT confirming the resolution of the lesion and intraoral examination revealing normal mucosa without swelling, underscoring the efficacy of conservative surgical management in this presentation.

## Conclusions

CGCG is a rare, benign intraosseous lesion with variable biological behavior, requiring careful clinical, radiographic, and histopathological correlation for accurate diagnosis. Although uncommon in the posterior maxilla, early diagnosis and tailored surgical intervention can yield excellent outcomes. In the present case, complete excision with curettage and peripheral ostectomy led to satisfactory healing with no recurrence at one year. Regular follow-up is essential, as most recurrences occur within the early years. This case emphasizes the importance of a structured diagnostic approach and supports conservative surgical management as a reliable treatment option for non-aggressive CGCG.
